# Association between human leukocyte antigen E expression and outcomes in solid tumors: a systematic review and meta-analysis

**DOI:** 10.3389/fonc.2025.1525924

**Published:** 2025-02-24

**Authors:** Javier David Benitez Fuentes, Jorge Bartolome Arcilla, Antonio David Lazaro Sanchez, Alicia de Luna Aguilar, Kauzar Mohamed Mohamed, Kissy Guevara-Hoyer, Pablo Ballestin Martinez, Miguel Borregon Rivilla, Asia Ferrandez Arias, Silvia Sánchez-Ramon, Alberto Ocaña

**Affiliations:** ^1^ Department of Medical Oncology, Elche General University Hospital, Alicante, Spain; ^2^ Department of Medical Oncology, Hospital Clinico San Carlos, Instituto de Investigación Sanitaria San Carlos (IdISSC), and CIBERONC, Madrid, Spain; ^3^ Experimental Therapeutics in Cancer Unit, Instituto de Investigación Sanitaria San Carlos (IdISSC), Madrid, Spain; ^4^ Department of Medical Oncology, Santa Lucia General University Hospital, Cartagena, Spain; ^5^ Department of Medical Oncology, Hospital General Universitario Morales Meseguer, Murcia, Spain; ^6^ Department of Immunology, IML and IdISSC, Hospital Clinico San Carlos, Madrid, Spain; ^7^ Department of Immunology, Ophthalmology and ENT, School of Medicine, Complutense University, Madrid, Spain; ^8^ Department of Medical Oncology, Hospital 12 de Octubre, Madrid, Spain; ^9^ START Madrid-Fundación Jiménez Díaz (FJD) Early Phase Program, Fundación Jiménez Díaz Hospital, Madrid, Spain

**Keywords:** human leukocyte antigen, HLA-E, cancer, solid tumors, survival, immunotherapy

## Abstract

**Background:**

Immunotherapy has gained momentum with the discovery of novel antibodies targeting immunosuppressive proteins. HLA-E, a non-classical major histocompatibility complex class I (MHC-I) protein, exhibits immunosuppressive properties, potentially influencing tumor immune evasion mechanisms. The association between Human Leukocyte Antigen E (HLA-E) expression and outcomes in solid tumors remains unclear.

**Methods:**

A systematic review of MEDLINE, Scopus, and the Cochrane Library up to March 15, 2024, was conducted following the PRISMA guidelines. Studies investigating HLA-E expression in solid tumors and its association with OS and DFS were included. Statistical analysis was performed using Comprehensive Meta-Analysis (version 3.0) with random-effects models.

**Results:**

After screening 657 articles, 11 studies were included, comprising a total of 1781 patients. The studies encompassed a variety of cancer types, follow-up periods, and staging details, with the majority focusing on non-metastatic cases. Notably, three studies evaluated colorectal cancer, while others focused on pancreatic, esophageal, brain, renal cell, gastric, endometrial, cervical, and hepatocellular carcinomas. The mean age of the patients was 59.81 ± 2.01 years, and the median follow-up period was 57.45 ± 8.91 months. HLA-E expression demonstrated no statistically significant association with OS (HR 0.913, 95% CI = 0.567-1.469; P=0.707), with significant heterogeneity observed (I2 = 84%). However, HLA-E non-expression was significantly associated with improved DFS (HR 1.406, 95% CI = 1.027-1.930; P=0.03), with moderate heterogeneity (I2 = 45%).

**Conclusion:**

This systematic review highlights that HLA-E expression in solid tumors could be a biomarker of better prognosis, measured by DFS. These findings align with the clinical benefit observed for agents targeting this pathway. However, further studies should be performed to confirm these preliminary observations.

**Systematic review registration:**

https://www.crd.york.ac.uk/prospero/display_record.php?ID=CRD42024527598, identifier CRD42024527598.

## 1 Introduction

In parallel with the development of personalized precision oncology treatments targeting key relevant genomic alterations, strategies acting on the immune system have demonstrated significant clinical activity (1). The approved therapies in this field target immunosuppressive proteins, therefore inducing the activation of the immune system (2). Among the different components of the immune system, human leukocyte antigen (HLA) proteins, including both class I and class II molecules, are key participants in immune regulation and immune escape in the cancer process (2, 3).

There are two major classes of HLA molecules: class I and class II. Class II HLA molecules, including HLA-DQ, HLA-DR, and HLA-DP, are primarily expressed on antigen-presenting cells (APCs) such as dendritic cells, macrophages, and B cells. These molecules play a critical role in presenting extracellular antigens to CD4+ T helper cells, thereby initiating and modulating adaptive immune responses. Dysregulation of HLA class II expression has been implicated in immune evasion by tumors, as reduced expression can impair the activation of CD4+ T cells and weaken antitumor immunity (3).

Class I HLA molecules are further divided into classical and non-classical subtypes (2). Classical type I HLA molecules (HLA-A, HLA-B, and HLA-C), are essential for immunosurveillance and cancer immunotherapy as T cells are presented with cellular antigens by this subtype (2, 4, 5). The non-classical HLA molecules, HLA-E, HLA-F, and HLA-G have immunosuppressive properties in contrast to traditional HLA-I molecules (2, 4, 5). HLA-E, found on chromosome 6, is formed by the association of a heavy chain and β2-microglobulin. This molecule presents peptides derived from the leader sequences of other MHC class I molecules, as well as potentially from pathogen-derived peptides (6). Its primary role is to enable Natural Killer (NK) cells to monitor the expression of the other class I HLA molecules, limiting NK action while also inhibiting T-cell cytotoxicity (6). Thus, HLA-E serves as a critical link between memory-driven adaptive immunity and the rapid response of innate immunity (5, 7–9).

Although it is frequently prevalent in trophoblast and tumor cells, HLA-E can be expressed at low levels in all nucleated cells (7, 10). Growing evidence indicates that tumors can use HLA-E expression to evade NK cell detection even in the absence of conventional class I HLA (8). A poor prognosis has been linked to HLA-E expression in various malignancies including colorectal cancer, gastric cancer, gynecological cancers, non-small cell lung cancer (NSCLC) and breast cancer (11–16).

Additionally, the tumor microenvironment (TME) is a dynamic ecosystem comprising immune cells, stromal components, and signaling molecules that collectively influence tumor progression and therapeutic responses (17–20). Within this setting, HLA-E can further facilitate immune evasion by engaging inhibitory receptors on NK and T cells, underscoring its relevance in shaping antitumor immunity and response to immunotherapies. In certain tumors, HLA-E expression can diminish the protective role of tumor-infiltrating lymphocytes, thus contributing to immune escape (20). Re-education strategies targeting the pro-tumor TME may help restore antitumor immune functions (17). This evolving understanding highlights HLA-E as a potential therapeutic target, particularly in combination with immunotherapies aimed at counteracting T cell exhaustion and enhancing treatment outcomes (18, 19).

HLA-E has shown significant therapeutic potential due to its expression in tumor cells compared to healthy tissues (21). By inhibiting cytotoxic NK cells and a subset of CD8 T lymphocytes through interaction with the NKG2A/CD94 heterodimer, it presents a promising target for novel therapeutic antibodies currently under evaluation in clinical studies (21). Targeting this pathway is vital because NK cells perform cytotoxic functions, facilitate antibody-mediated cellular cytotoxicity (ADCC), and recruit dendritic cells into tumors enhancing communication between the innate and adaptive immune systems (21).

Considering the importance of this family of proteins and particularly the relevant role of HLA-E for therapeutic purposes we conducted a systematic review and meta-analysis to investigate the predictive relationship between HLA-E and survival in solid tumors.

## 2 Material and methods

This systematic review and meta-analysis were performed in accordance with the Preferred Reporting Items for Systematic Reviews and Meta-analysis (PRISMA) (22) and was registered in PROSPERO (CRD42024527598).

### 2.1 Literature search and study selection

A comprehensive search was performed on MEDLINE, Scopus, and the Cochrane Library from inception till 15^th^ March 2024. The following keywords were used in the search string, along with Boolean Operators “AND” and “OR” to design an encompassing search string: “human leukocyte antigen E” or “HLA-E”, “solid tumors”, “neoplasm”, “malignancy”, “cancer” and “clinical outcomes” or “prognosis.” Additionally, bibliometrics of published articles, conference abstracts and grey literature were reviewed to ensure there were no missing articles.

Articles were screened to identify and remove the duplicates. The remaining articles were then revised by independent reviewers (JDBF, JBA, ADLS, ALA, KMM), to ensure that the recruited articles met the defined eligibility criteria. The inclusion criteria used to shortlist studies were: (i) Studies published in English language, (ii) Observational, cross-sectional, cohort or randomized controlled trials, (iii) Studies involving human participants diagnosed with solid tumors, (iv) Studies reporting at least one of the outcomes of interest.

### 2.2 Data extraction and quality assessment

Five independent reviewers (JDBF, JBA, ADLS, ALA, KMM) conducted the data extraction and verification process. Any disagreements were settled through discussion and the input of a third reviewer (AO). The data gathered from each study encompassed several variables: the study population and the year of publication, the sample size, basic patient demographics (age and gender), tumor type, median follow-up period in months, the method of HLA-E detection (in tissue versus plasma), the detection techniques employed (such as Immunohistochemistry [IHC], Enzyme-Linked Immunosorbent Assay [ELISA], or mRNA analysis), the specific antibody used for detection (when applicable), the percentage of patients exhibiting HLA-E expression, and the outcomes reported by the studies. The primary outcomes analyzed included Overall Survival (OS) and Disease-Free Survival (DFS).

### 2.3 Statistical analysis

Comprehensive Meta-Analysis (CMA) version 3.0 was used for all relevant meta-analyses of this study. A random-effects model was used due to variability in study populations, differences in study designs and methods, and unknown sources of heterogeneity. Publication bias was assessed by checking asymmetry in the funnel plots generated via CMA. A p-value <0.05 was considered statistically significant for all outcomes. Heterogeneity was assessed with Higgin’s I^2^ test. A value of I^2^ = 25%-50% was considered mild, 50%-75% as moderate, and >75% as significant heterogeneity.

## 3 Results

### 3.1 Literature search

**Figure 1:**
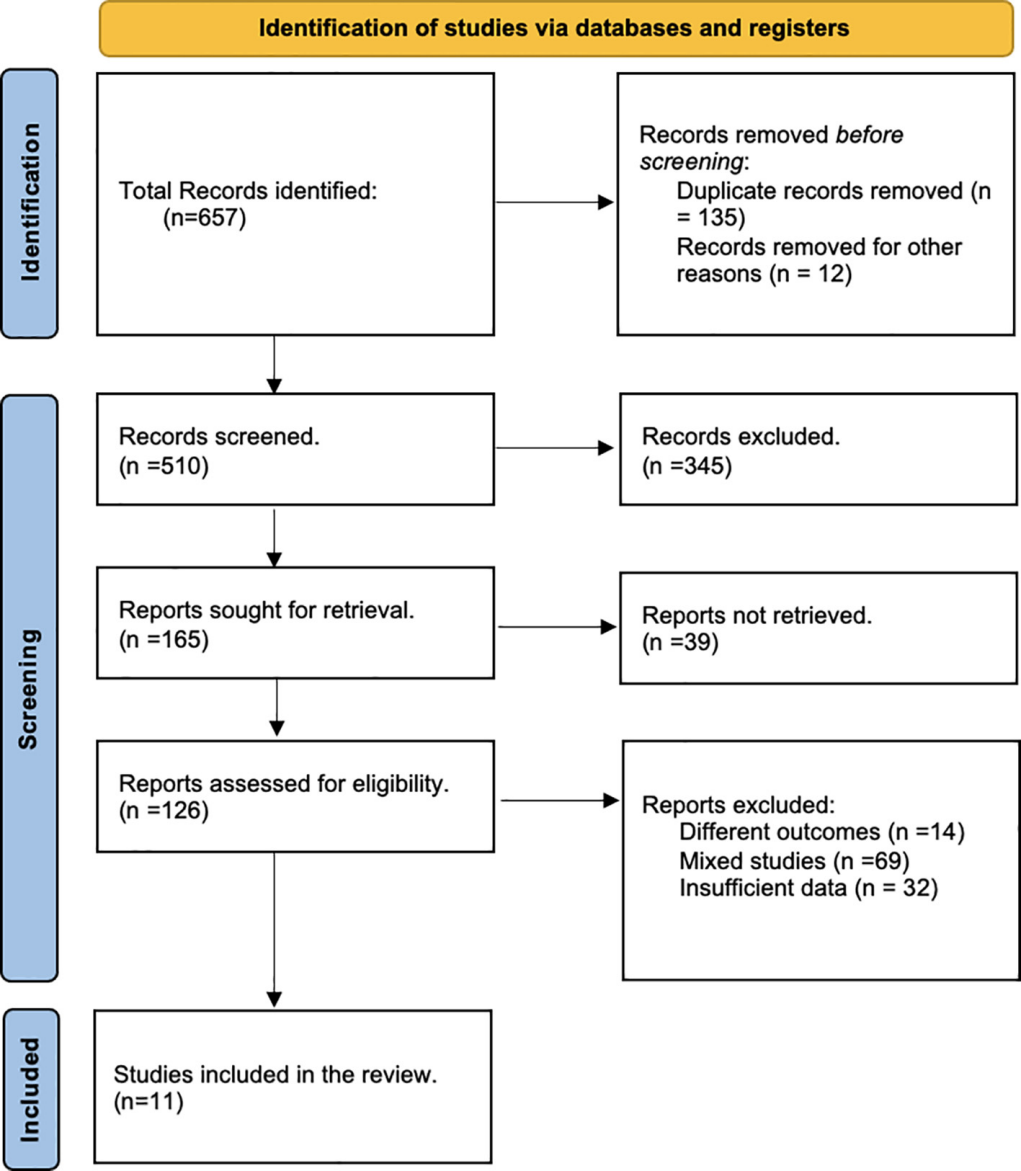
Preferred Reporting Items for Systematic Reviews and Meta-Analyses (PRISMA) flow chart of literature search.

A total of 657 articles were identified from the literature search (**Figure 1**). After removal of duplicates as well as reviews, abstracts, editorials, and case reports, 126 studies were given a full-text evaluation, and 11 were finally included in the meta-analysis (11–13, 23–30).

### 3.2 Study characteristics

A total of 1781 patients were included across the 11 studies, with their characteristics summarized in **Table 1**. Three studies evaluated colorectal cancer (11, 26, 28), while others included pancreatic (23), esophageal (24), brain (25), renal cell (27), gastric (12), endometrial (29), cervical (13), and hepatocellular carcinoma (30). The mean age of the patients was 59.81 ± 2.01 years. The median follow-up for the studies was calculated to be 57.45 ± 8.91 months. Four of the studies were conducted in China (24–27), while three in Japan (12, 23, 30). Two studies were conducted in Italy (11, 28) as well as two in the Netherlands (13, 29). Staging information was heterogeneous across the studies with varying levels of detail provided. None of the studies offered a comprehensive account of any systemic therapies administered.

**Table 1:** Characteristics of included studies.

Study	Country	Patients (N)	F (%)	Mean Age (years)	Cancer	Stage (%)	FU (months)	Outcome reported
Hiraoka 2020 (23)	Japan	98	36.1	60	Pancreatic Cancer	M0 89.8, M1 10.2	17.6	OS and DFS
Guo 2015 (26)	China	137	43.3	60	Colorectal Cancer	M0 94.1, M1 5.9	NA	OS
Xu 2021 (24)	China	110	30.9	58	Esophageal Cancer	NA	60	OS
Levy 2008 (11)	Italy	42	38	66	Colorectal Cancer	Dukes’ B 42.9, Dukes’ C 57.1	NA	DFS
Wu 2020 (25)	China	261	40.2	50.5	Diffuse Glioma	NA	54.9	OS and DFS
Chu 2020 (27)	China	55	NA	NA	Renal Cell Carcinoma	NA	NA	OS
Morinaga 2022 (12)	Japan	232	22.1	NA	Gastric Cancer	Stage I 17.7, Stage II 42.7, Stage III 39.6	32.8	DFS
Benevolo 2011 (28)	Italy	149	52.3	64	Colorectal Cancer	Stage II 65.8, Stage III 34.2	71.5	OS and DFS
Versluis 2017 (29)	Netherlands	335	100	60	Endometrial Cancer	NA	72	DFS
Gooden 2011 (13)	Netherlands	150	100	NA	Cervical Cancer	Early Stage 100	NA	OS
Wang 2019 (30)	Japan	212	14	60	HCC	BCLC Stage 0 9.4, Stage A 52.8, Stage B 26.4, Stage C 11.3	NA	OS and DFS

DFS, Disease free survival; F, Female; FU, mean follow-up; HCC, Hepatocellular Carcinoma; M0, non-metastatic; M1, metastatic; OS, Overall Survival.

The HLA-E expression percentage was calculated to be approximately 70.23% across all reported studies. All articles used tissue samples for HLA-E detection, predominantly employing IHC as the 1 technique. **Table 2** details the characteristics of antibodies and their detection methods used in each study. The MEM-E/02 antibody was consistently reported across most studies (11–13, 23, 24, 26, 28, 29) except for three studies that used mRNA-based detection methods (25, 27, 30).

**Table 2:** Summary of the detection methods and HLA-E expression percentages in the included studies.

Study	HLA-E positive %	HLA-E Detection Type	HLA-E Detection Technique	HLA-E Detection Antibody	Positivity Threshold/Criteria
Hiraoka 2020 (23)	NA	Tissue	IHC	MEM-E/02	Staining >10% tumor cells
Guo 2015 (26)	65.7	Tissue	IHC	MEM-E/02	Staining observed in tumor cells
Xu 2021 (24)	88.2	Tissue	IHC	MEM-E/02	Staining >5% tumor cells
Levy 2008 (11)	75	Tissue	IHC	MEM-E/02	Staining in tumor cells ≥75th percentile
Wu 2020 (25)	NA	Tissue	mRNA microarray analysis	Not applicable	HLA-E mRNA expression > median value of the group
Chu 2020 (27)	NA	Tissue	mRNA expression analysis	Not applicable	NA
Morinaga 2022 (12)	41.3	Tissue	IHC	MEM-E/02	Positive if score (staining intensity [0–3) multiplied by percentage of positive tumor cells (0–4]) ≥1
Benevolo 2011 (28)	NA	Tissue	IHC	MEM-E/02	Staining ≥70% tumor cells
Versluis 2017 (29)	75	Tissue	IHC	MEM-E/02	Positive if score (staining intensity [0–3) multiplied by percentage of positive cells (0–5]) ≥2.5
Gooden 2011 (13)	76.2	Tissue	IHC	MEM-E/02	Positive if score (staining intensity [0–3] multiplied by percentage of positive cells [0–5]) ≥5
Wang 2019 (30)	NA	Tissue	mRNA expression analysis	Not applicable	HLA-E mRNA expression > median value of the group

IHC, Immunohistochemistry; NA, Not Available.

### 3.3 Overall survival

**Figure 2:**
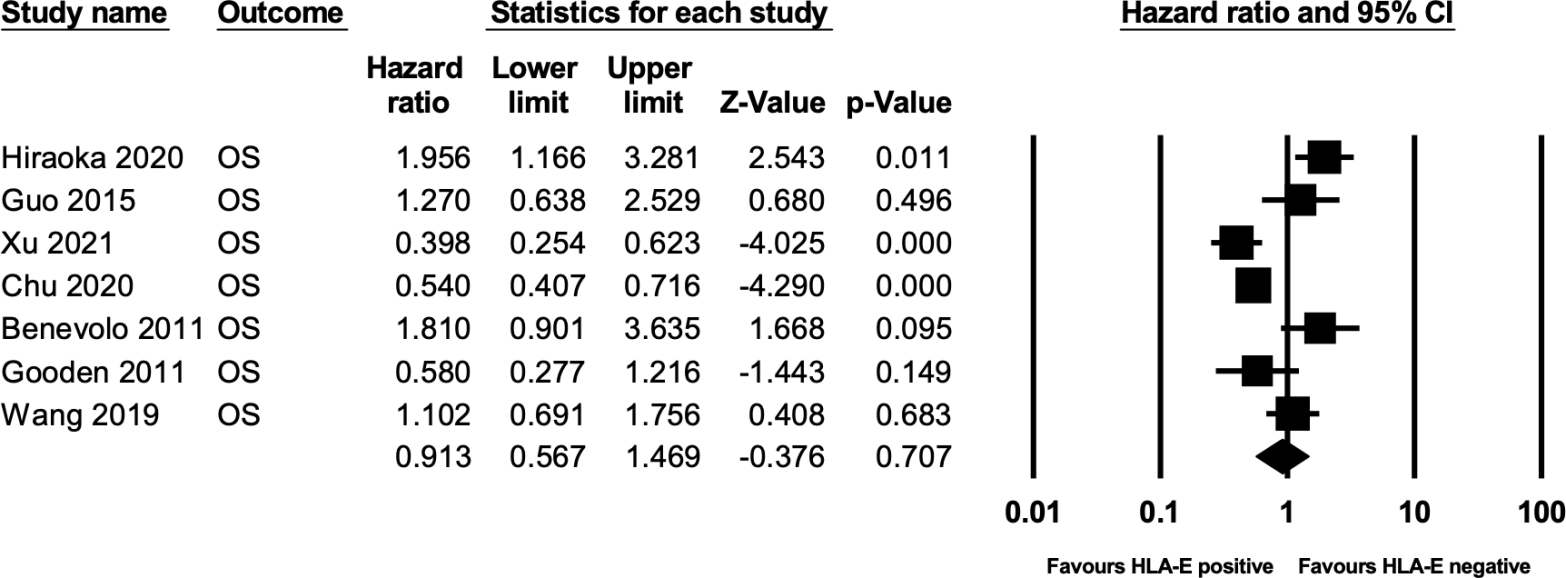
Forest plot of OS.

Seven studies (13, 23, 24, 26–28, 30) comprising 911 patients reported HRs for OS, the forest plot of the outcome is illustrated in **Figure 2**. Our combined analysis demonstrated that HLA-E expression had no statistically significant relationship with OS (HR 0.913, 95% CI = 0.567-1.469; P=0.707). Heterogeneity was calculated via a fixed-effect model and hence was demonstrated to be significant. (I2 = 84%).

### 3.4 Disease free survival

**Figure 3:**
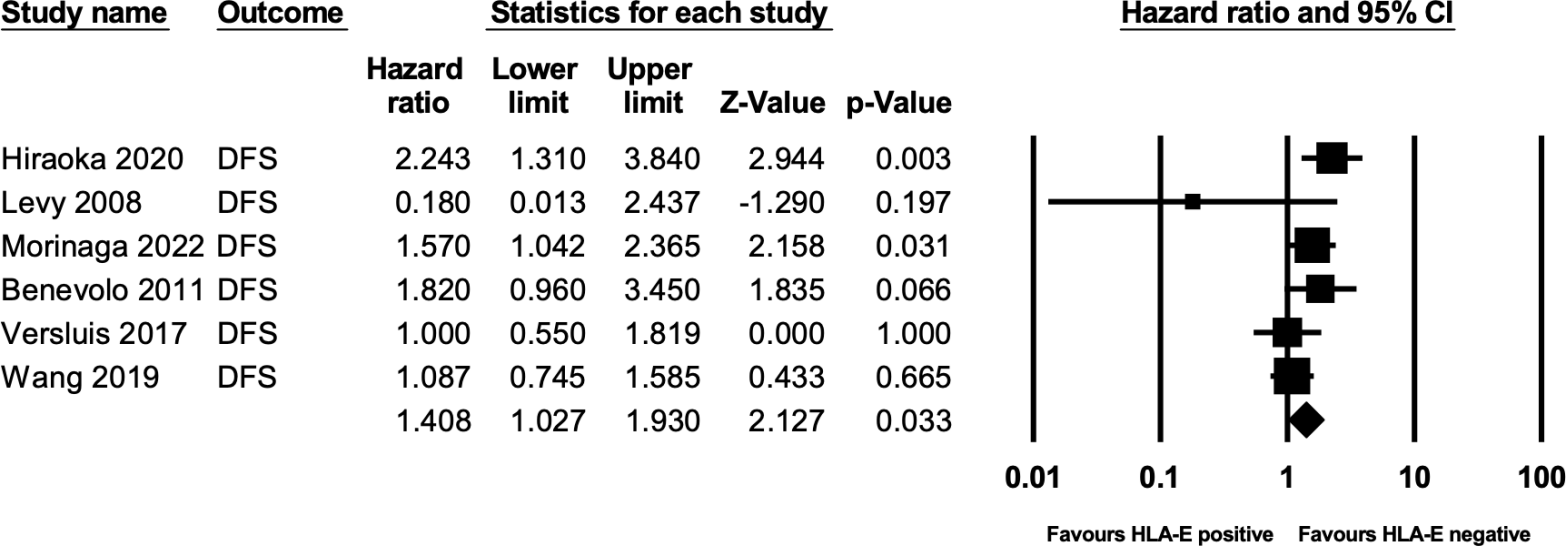
Forest plot of DFS.

A total of six studies (11, 12, 23, 28–30) comprising 1068 patients reported HRs for DFS. The forest plot of the outcome is illustrated in **Figure 3**. On analysis via a random effect model, we determined that HLA-E non-expression was associated with disease-free survival, and it was statistically significant (HR 1.406, 95% CI = 1.027-1.930 9; P=0.03). Heterogeneity was moderate amongst the given studies (I2 = 45%).

## 4 Discussion

HLA-E expression levels, typically elevated in tumor cells compared to healthy tissues, lead to the inhibition of cytotoxic NK cells and CD8 T lymphocytes through interaction with the NKG2A/CD94 heterodimer (2, 21, 31, 32). Consequently, tumor cells may evade NK cell action through HLA-E overexpression (21, 33). This immunosuppressive mechanism emphasizes the potential therapeutic importance of targeting the NKG2A/HLA-E axis (13, 15, 21, 34). This systematic review and meta-analysis investigated the relationship between HLA-E expression and survival in solid tumors. Our analysis revealed no statistically significant association between HLA-E expression and OS. However, HLA-E non-expression was significantly associated with improved DFS. The included studies encompassed a diverse range of solid tumors, with considerable variability in staging and follow-up periods.

**Figure 4:**
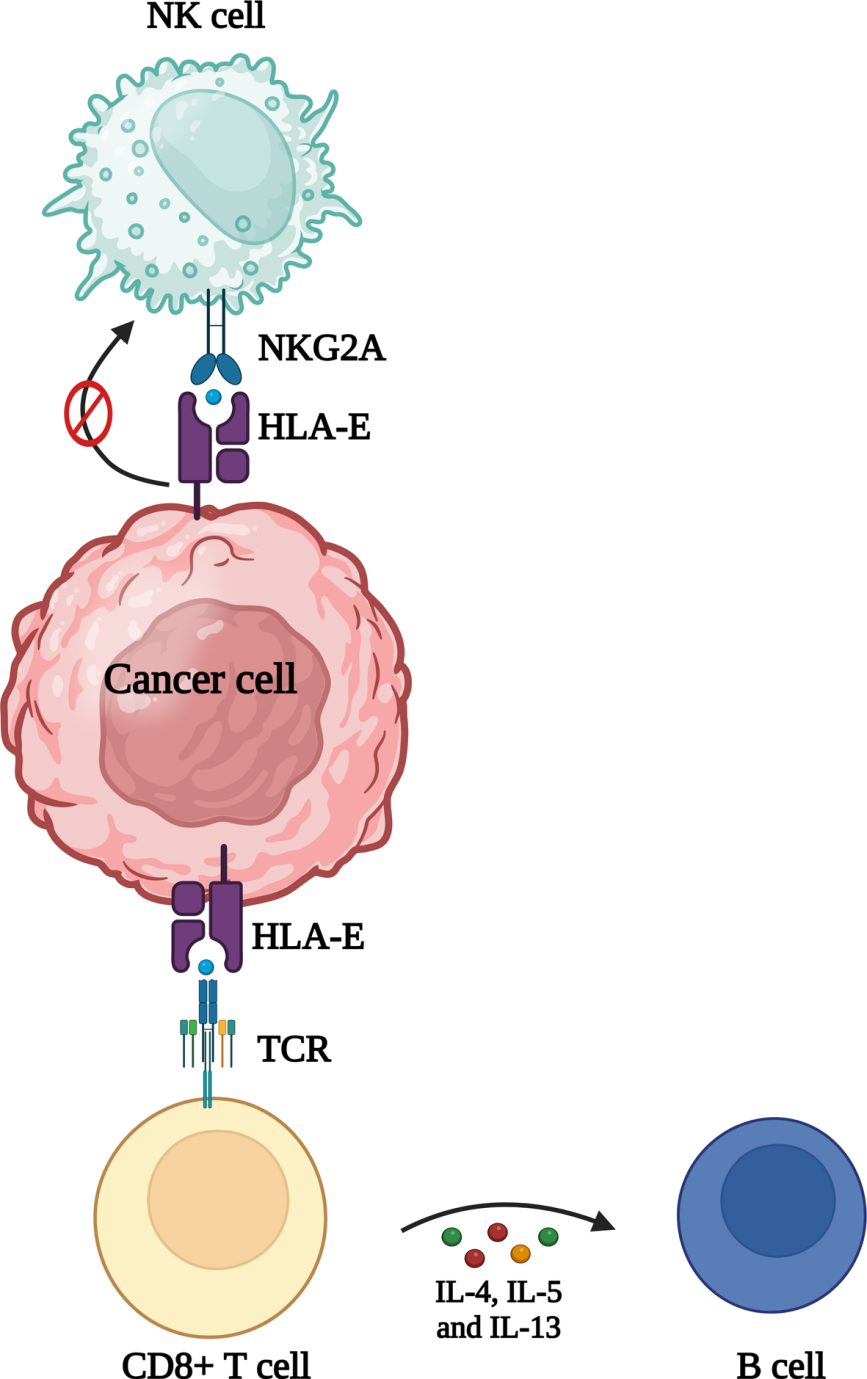
Role of HLA in cancer. Overexpression of HLA-E is frequently observed in cancer cells and can contribute to evasion of NK cell and CD8^+^ T cell mediated detection and lysis. Additionally, promoting a Th2 response might reduce the cytotoxic potential of CD8^+^ T cells, aiding in immune evasion.

HLA-E binding peptides are primarily responsible for controlling HLA-E expression (31, 32). Tumors may upregulate the expression of HLA-E by ensuring a sufficient supply of peptides that stabilize HLA-E, thereby engaging the inhibitory receptor NKG2A on NK cells and certain T cells to protect themselves from immune attack (35, 36). HLA-E can modulate CD8+ T cell responses, and under certain conditions, can induce a Th2-like cytokine profile and activate B cells (**Figure 4**) (37, 38).

HLA-E exists in different conformational forms, notably the peptide-bound form and the open conformer. The peptide-bound form, associated with β2-microglobulin and a peptide ligand, is the predominant form expressed on the cell surface and interacts with inhibitory receptors such as NKG2A/CD94 on NK cells and CD8+ T cells (36). In contrast, the open conformer lacks a bound peptide and may have altered stability and receptor interactions (39). The conformational state of HLA-E can influence its expression levels, immune recognition, and function in immune regulation (36, 39).

Depending on the receptor and the responding cell, HLA-E engagement can result in immune activation or suppression (31, 40). As a result, several studies have investigated the connection between HLA-E expression and tumors in recent years. Patients with melanoma (41), glioblastoma (42), gastric cancer (12), breast cancer (16), and ovarian cancer (32) were reported to have high expression of HLA-E and the expression level of HLA-E was linked to clinical outcomes. Another study reported a similar correlation between HLA-E expression and disease-free survival in bladder cancer patients (43).

In the current study, HLA-E made no difference in OS. The reasons for the disparity of the results could be due to different histologies as well as differences in methodology. Our results for DFS, however, showed that overexpression of HLA-E was associated with a poor DFS. Across the six studies included in our DFS analysis, a common theme emerged: HLA-E expression was linked to worse DFS outcomes, despite the variability in cancer types and methodologies. *Levy* et al. evaluated colorectal cancer patients using IHC, finding that high HLA-E expression was significantly associated with shorter DFS, especially in Dukes’ C patients (11). *Levy* et al. also highlighted the role of HLA-E in immune evasion through NK cell inhibition (11). Similarly, *Morinaga* et al. (12), *Hiraoka* et al. (23), *Benevolo* et al. (28), and *Versluis* et al. (29), found that higher HLA-E expression correlated with poorer DFS across esophageal squamous cell carcinoma, pancreatic ductal adenocarcinoma, cervical cancer, and endometrial cancer. Lastly, *Wang* et al. in their mRNA expression analysis in hepatocellular carcinoma patients showed that higher HLA-E mRNA levels were associated with worse DFS (30). In this context, in primary tumors, predominantly from early-stage disease, HLA-E could play a key immunosuppressive role (31) and therefore could be associated with a detrimental prognosis. In line with their potential role as a therapeutic target, treatments targeting their receptor, NKGD2, are currently under research (44).

Therapeutic strategies targeting the NKG2A/HLA-E axis include both monotherapies and combination therapies (21, 45). While direct inhibition of HLA-E could potentially prevent its interaction with the activating NKG2C receptor, the preferred approach has been to block NKG2A (46). Monalizumab, a first-in-class humanized IgG4 antibody targeting the NKG2A/CD94 receptor, has shown promise in preclinical studies by enhancing NK and CD8 T cell activity (44, 45). Monalizumab blocks the interaction of NKG2A/CD94 with HLA-E, thus promoting NK and CD8 T cell activation. This mechanism not only enhances cytotoxic activities but also augments antibody-dependent cellular cytotoxicity (ADCC), a crucial effector function for many anticancer antibodies (46, 47). Early clinical trials with Monalizumab alone or in combination with anti-PD-L1 antibodies like Durvalumab have demonstrated acceptable safety profiles and preliminary signs of immune activation, the COAST study reported improved overall response rates (ORR) and progression-free survival (PFS) with Monalizumab and Durvalumab in unresectable stage III NSCLC patients (48).

Clinical trials have also explored various combinations of Monalizumab with other therapeutic agents in the recurrent or metastatic setting (46, 49, 50). Other monoclonal antibodies like S095029 and HY-0102 are in clinical development (51, 52). However, tumors may compensate for NKG2A/HLA-E blockade by upregulating other immunosuppressive molecules like PD-L1 or IDO, thereby preserving an immune-evasive niche (18, 20).

To the best of our knowledge, our study is the first meta-analysis to discuss HLA-E and its role in the outcomes of solid cancer patients, including OS and DFS. It offers valuable insights into the prognostic significance of HLA-E expression in various solid tumors. However, to contextualize the interpretation of our findings, it is important to highlight several limitations. A significant limitation is the pronounced heterogeneity among the included studies, encompassing diverse tumor types (e.g., colorectal, pancreatic, esophageal, brain, renal cell, gastric, endometrial, cervical, and hepatocellular carcinomas) and varying disease stages. Each of these cancer types possesses unique biological behaviors, prognostic factors, treatment modalities and responses to treatment. The variability in cancer stages across studies further compounds this issue. This heterogeneity in tumor types and stages may confound the relationship between HLA-E expression and survival outcomes, thereby limiting the ability to generalize our findings. There was substantial variation in the methods used to quantify HLA-E expression. While the majority of studies employed IHC to detect HLA-E levels in tissue samples, others utilized mRNA expression analyses, and the positivity thresholds can varied widely. Importantly, the specificity of the MEM-E/02 antibody for HLA-E detection warrants consideration, as potential cross-reactivity with other HLA class I molecules or recognition of different conformational forms of HLA-E could impact the accuracy of HLA-E expression assessment (53). Such discrepancies in detection techniques, antibody specificity, and positivity criteria can lead to inconsistencies in categorizing patients as HLA-E positive or negative, potentially impacting the results. Moreover, our study did not correlate HLA-E expression with T cell and NK cell infiltration or the expression of HLA-E-specific receptors on immune cells, which limits our understanding of the immunological context and mechanisms of immune evasion (31). Furthermore, HLA-E expression can be influenced by underlying polymorphisms, such as the commonly studied HLA-E*01:01 and HLA-E*01:03 alleles, which differ in expression levels and surface stability (8, 35, 43). These variations may modulate immune recognition and the extent of immune evasion within the TME, further highlighting the complexity of HLA-E’s prognostic and therapeutic role. Much of the research that comprises our analysis is retrospective, which brings with it inherent limitations including selection bias, dependence on pre-existing data sources and confounding variables. Additionally, relying solely on published literature could introduce publication bias. These limitations could affect the validity of our findings. Although the results of our meta-analysis shed light on the predictive importance of HLA-E expression in solid tumors, they do not clarify the underlying mechanisms responsible for these correlations. To shed light on the causative pathways and biological significance of our findings, further research is necessary to fully understand the intricate interactions between HLA-E expression, tumor biology, immune microenvironment, and treatment modalities.

Our systematic review and meta-analysis provide comprehensive insights into the prognostic significance of HLA-E expression in solid tumors. Despite the lack of statistical significance in the relationship between HLA-E expression and OS, our results point to a possible predictive role for HLA-E non-expression for improved DFS suggesting that targeting the NKG2A/HLA-E axis could be a valuable strategy in enhancing antitumor immune responses, which could be particularly beneficial for early-stage diseases. However, several challenges remain. The variability in clinical outcomes highlights the need for more research to optimize treatment combinations and identify the patient populations that would benefit most from NKG2A/HLA-E axis inhibition. In particular, prospective studies and clinical trials evaluating HLA-E as a therapeutic target will be instrumental. Moreover, understanding the underlying mechanisms of immune evasion and the role of HLA-E in different tumor microenvironments is crucial for developing effective treatments. Further investigation is essential to develop these approaches, optimize combination strategies, and elucidate the mechanisms driving the variable clinical outcomes across different cancer types and stages.

## Data Availability

The datasets generated during the current study are not publicly available due to local research institute and department regulations. Requests to access these datasets will be made available by the corresponding author upon reasonable request.
